# Content of Cadmium and Nickel in Soils and Assimilatory Organs of Park Woody Species Exposed to Polluted Air

**DOI:** 10.3390/life12122033

**Published:** 2022-12-05

**Authors:** Ivica Pivková, Ján Kukla, Helena Hniličková, František Hnilička, Danica Krupová, Margita Kuklová

**Affiliations:** 1 Institute of Forest Ecology, Slovak Academy of Sciences, 960 01 Zvolen, Slovakia; 2 Department of Botany and Plant Physiology, Czech University of Life Sciences Prague, 165 00 Prague, Czech Republic; 3 National Forest Centre—Forest Research Institute, T. G. Masaryka 22, 960 92 Zvolen, Slovakia

**Keywords:** park objects, trees, leaves, needles, Fluvisol, Phaeozem, risk elements, contamination factors

## Abstract

The rising level of pollutant emissions is becoming one of the most pressing environmental problems of our time. Therefore, this work is focused on evaluating Cd and Ni contamination of soils and assimilatory organs of two native (*Acer platanoides* L., *Taxus baccata* L.) and two non-native (*Negundo aceroides* Moench, *Thuja occidentalis* L.) woody species in urban parks of SW Slovakia. The contents of Cd and Ni in soils were determined by the AAS method and, in the assimilatory organs of trees, by the AAS-ETA method. The studied soils (Fluvisol, Phaeozem) have neutral soil reactions and a moderate organic matter content. Cadmium soil contamination is considerable to very high; in the case of Ni, it is moderate to low. Cadmium levels detected in leaves were 31% higher than in needles, while Ni levels were 27% lower. Significant ecological factors in relation to the studied woody species were evaluated using PCA. The first three principal components of PCA significantly correlated with Cd (PC1) and Ni (PC3) contents in soils and Cd content in assimilatory organs (PC2), thus suggesting that these elements could especially originate from industrial and vehicular sources. Knowledge of the factors affecting the accumulation of risk elements in the assimilatory organs of park woody species can be successfully used, especially in the assessment of the quality of the urban environment and the selection of suitable cultivars for planting in areas with air pollution.

## 1. Introduction

The growing level of pollutant emissions, especially in urban areas, results in increased amounts of risk elements emitted into the atmosphere that are later deposited into the soil [1,2,3]. The impact of risk elements on the environment is accentuated by their non-degradability. Although heavy metals (HM) may occur naturally in soil, additional contributions to the environment can be derived mainly from anthropogenic sources such as agriculture, urbanization, industrialization, and mining [4]. That’s why elevated levels of HM, such as cadmium and nickel, in the environment are a reality today. Increasing concentrations of risk elements can cause serious environmental problems to soil, water, and biota. The constantly changing environment of cities and the surrounding landscape causes plant stress manifested by physiological growth disorders, destructive phenomena and changes in vegetative organs, loss of vitality, reduced life expectancy, and premature death of plants. Many plants are in a position to take up large quantities of certain chemical elements from the environment [5,6]. In fact, the foliage of tree species is the main sink for many pollutants, and therefore they are more sensitive to their effects than other plant organs [7]. This particularly occurs when plants are exposed to massive environmental pollution of atmospheric origin.

Cadmium has no known physiological function in plants and can be toxic. It is released into the environment by power stations, heating systems, metal-working industries, or urban traffic [8]. Zhao et al. [9] report that in urban soils around the Tanggu industrial district in Tianjin (China), Cd is mainly controlled by industrial discharges, sewage sludge, and municipal solid waste. As one of the most toxic environmental pollutants, Cd has a strong influence on the metabolic activities of crop plants by inducing several physiological changes and growth inhibition [10]. Even at relatively low concentrations, it can exert strong toxic effects on crops [11]. In general, the uptake of Cd can vary greatly among plant species and also among cultivars within a species [12]. For example, the distribution of Cd in different growth stages of *Tlaspi caerulescens* varied according to the plant organ [13]. Xu et al. [14] found that leafy vegetables cultivated in gardens and suburban areas of southern China contain excessive amounts of Cd (0.21 to 0.99 mg kg^−1^) and could therefore threaten human health.

Nickel is considered to be essential for plant growth at low concentrations; however, Ni pollution is increasing in the environment, and therefore, it is important to understand its toxic effects on plants [15]. It is usually found in higher concentrations only in the surroundings of Ni works and coal and oil-burning power stations. Pacyna and Pacyna [16] report that almost 90% of the global anthropogenic Ni emissions originate from oil combustion. It becomes toxic to plants that have absorbed higher than Ni threshold values [17]. Although root uptake is the main pathway for Ni access in higher plants, there is much evidence showing that Ni is available to plants also through foliage [18,19].

Trees as ecological indicators have mostly been used for the biological monitoring of urban air quality [20]. Plant leaves are useful indicators of pollutants when evaluating air pollution because they have a high distribution density [21]. The use of leaves for assessing vegetation stress levels and toxicity states has numerous ecological benefits because trees, as long-lived organisms, reflect mainly the cumulative effects of environmental pollution of soil and atmosphere pollution [22]. There are several examples of trees used as biomonitors for air and soil pollution, e.g., *Pinus sylvestris* in Poland [23], *Populus nigra* in Bulgaria [24], *Populus alba* in south Spain [22], as well as several tree species of urban environments in Greece [25]. For example, *Acer pseudoplatanus* L. has been used as a bioindicator for assessing air contamination in urban ecosystems in Europe [26], and *Quercus ilex* L. has been used as a bioaccumulator for HM in urban areas in Italy [27].

This contribution is focused on the study of the impact of environmental load on the assimilatory organs of two native (*Acer platanoides* L. and *Taxus baccata* L.) and two non-native (*Negundo aceroides* Moench and *Thuja occidentalis* L.) woody plants in the urban environment of SW Slovakia. Native woody species are often considered more efficient in terms of growth, reproduction, and survival under environmental stress [28]. Therefore, there is an ongoing interest in searching for plant species that are tolerant to HM and can be used to remove them from the soil through root absorption and accumulation in shoots and leaves. Non-native woody species were often imported as ornamental or honey-bearing plants. Some of them can spread quickly from parks and plantations to the surroundings and occupy new areas. Conifers are frequently used as bioaccumulators in environmental research. As a rule, they are evergreen and rarely deciduous trees that can bind pollutants from the air for several years in their assimilation apparatus [29]. Some studies have shown that fast-growing trees can accumulate more HM from the soil than from the atmosphere [30]. According to Liang et al. [21], HM in plant needles come mainly from the soil, and the HM in leaves of broad-leaved plants results from atmospheric deposition.

We assume that the studied parks may be exposed to varying degrees of polluted air and soil by Ni and Cd because these elements are part of emissions from industrial plants in this area (production of aluminum profiles, chemical, food industry, agricultural production, and waste disposal). Transport, particle suspensions from insufficiently clean roads, construction sites, landfills for bulk materials, and chimneys of houses heated with solid fuels also significantly contribute to air pollution.

The main purpose of this study was to determine and evaluate the contents of Cd and Ni in the soil and assimilatory organs of selected native and non-native (coniferous and leafy) woody species located in urban parks of SW Slovakia from the aspect of their tolerance to the contaminated environment. The obtained results should contribute to understanding the relationships between the level of environmental pollution, the level of contamination in woody plants, and their ability to indicate the risk of environmental contamination in selected settlements.

## 2. Materials and Methods

### 2.1. Study Site

**Figure 1:**
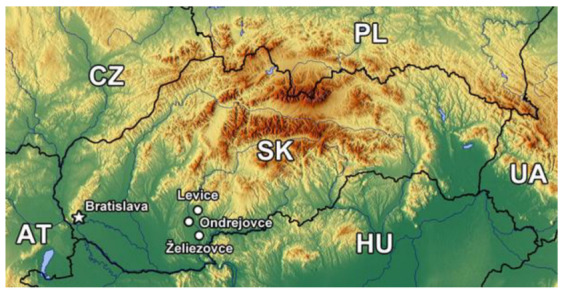
The location of the studied parks (Levice, Želiezovce, Ondrejovce) in SW Slovakia.

Location and basic information about studied park objects (PO) of SW Slovakia are presented in Figure 1 and Table 1. The investigated parks (Ondrejovce, Levice, and Želiezovce) are part of the agro-industrial Nitra region. The town of Želiezovce is located about 25 km south-southeast of the district city of Levice, and the village of Ondrejovce is about 17 km southwest of Levice city. The research was carried out during the summer aspect of phytocenoses from July to August 2012 and 2018. The vegetation of the parks in Levice and Želiezovce grows on Fluvisol, and on Phaeozem in the park in Ondrejovce.

According to the phytogeographic classification of Slovakia [31], historical parks belong to the area of the Pannonian flora (Pannonicum), the area of the Eupannonian xerothermic flora (Eupannonicum), and the Podunajská nížina district. From a phytogeographic-vegetation point of view, the parks are part of the oak zone, lowland subzone, undulating (hilly) area, Hronská niva district—southern subdistrict (Levice, Želiezovce), and the Hronská pahorkatina district—northern subdistrict (Ondrejovce). In terms of potential natural vegetation, these are ash-elm-oak forests in the basins of large rivers (hard floodplain forests) and Peripannonian oak-hornbeam forests [32].

There are 111 taxa of woody plants in Levice park (41% native, 59% introduced, 78% broadleaved, and 22% coniferous). Urban park in Želiezovce is the source of 98 taxa, of which 45% are native, 55% introduced, 80% broadleaved, and 20% coniferous. In the historical park of Ondrejovce, there were 52 taxa of woody plants (50% native, 50% introduced, 86% broadleaved, and 14% coniferous).

Assimilatory organs were sampled from two native (*A. platanoides*, *T. baccata*) and two non-native (*N. aceroides*, *T. occidentalis*) woody species. From an ecological point of view, *A. platanoides* is a semi-shade-loving woody plant, demanding soil moisture and sufficient soil nutrients. In Slovakia, it is a native tree frequently planted as an ornamental tree in parks. *N. aceroides* is a semi-shade-loving to light-loving woody plant that is undemanding on soil nutrients and is resistant to frost and urban environments. It comes from North America, where it is part of mixed forests. In Slovakia, the species is considered invasive. *T. baccata* is a shade-loving tree dependent on soil moisture. It is a native tree that tolerates polluted urban environments well. *T. occidentalis* comes from North America, where it forms homogeneous and mixed stands. It is a semi-light-loving and shade-loving woody plant, undemanding to the soil, that is resistant to frost and the urban environment.

### 2.2. Soil Analyses

In each park object, three undisturbed soil columns were randomly sampled using a linear soil sampler (Eijkelkamp) and subsequently divided in the laboratory into 0–5 cm, 10–20 cm, and 20–30 cm layers. A total of 9 samples were obtained, which, after air-drying, were sifted through a sieve with a mesh size of 2 × 2 mm. Sampling was carried out in 2012 and 2018, and the results of the analyses were averaged.

The active and exchange soil reactions were determined using a digital pH meter Inolab pH 720 (Weilheim, Germany), and the ratio of fine earth to water or 1 M KCl solution was 1:2.5. The content of carbonates in the soil (CaCO_3_ equivalent) was determined by the volumetric method using a calcimeter Ing. Janka (release of CO_2_ after application of 4 N hydrochloric acid solution). Carbon and nitrogen contents have been determined using CNS Flash EA 1112 from Thermo Finnigan (three replications were performed according to STN EN ISO 16948). The amount of humus was calculated by multiplying the carbon content by a factor of 1.724.

Subsamples of soils intended for the determination of Cd and Ni concentrations were ground down (<0.001 mm) using a Fritsch planetary micro-mill (Idar-Oberstein, Germany). Digestion of the samples using HNO_3_-H_2_O_2_ solution was performed in a MWS-2 microwave system in a speedwave MWS-2 system (Berghof, Germany). Cadmium and nickel contents of the filtrates were determined by the AAS method (STN ISO 8288) using a Thermo iCE 3000 Series AAS instrument (Thermo Scientific, Cambridge, UK). Soil names are listed according to the principles of the IUSS Working Group WRB [33]. The values of all studied soil parameters are based on the dry weight.

### 2.3. Analyses of Assimilatory Organs of Woody Plants

Assimilatory organs were randomly sampled from individuals of two native (*A. platanoides*, *T. baccata*) and two non-native (*N. aceroides*, *T. occidentalis*) woody species. In each park, samples of assimilatory organs were taken from 3 randomly selected individuals of each woody species (4 × 3 = 12 trees/park). Approximately 50 leaves or 50 conifer shoots (15 cm long) were collected from each tree in triplicate. The samples of the assimilatory organs were taken from approximately 2 to 3 m above the ground, from the N, W, and S sides of the tree. The HM content was determined for each sample (3 trees × 3 samples = 9 samples/woody species).

The quality of the assimilatory organs of woody plants was controlled by their energy content. Plant samples were dried at 80 °C for 48 h and homogenized with a Fritsch planetary micro mill (˂0.001 mm). Powder samples in amounts of 0.7–1 g (pressed into a briquette) were dried to a constant weight at 100 °C and completely burned in an oxygen atmosphere in the calorimetric vessel of an IKA C-4000 adiabatic calorimeter (C-402 software, DIN 51900 standard, Heitersheim, Germany). Measured values of the heat of combustion (J g^−1^ in dry matter) were used to determine the gross energy in assimilatory organs of studied woody plants. The relative variation between measurements of each sample was limited to less than 1%.

Plant samples were washed carefully to remove dust particles, dried in an oven at 80 °C for 48 h, and homogenized using a Fritsch planetary micro mill PULVERISETTE 7 classic line (˂0.001 mm). Microwave digestion of samples with HNO_3_-H_2_O_2_ was performed using a speedwave MWS-2 system (Berghof, Germany). The contents of Cd and Ni in the filtrates were determined by the AAS-ETA method (STN ISO 11047) on a Thermo iCE 3000 Series AAS instrument (Thermo Scientific, Cambridge, UK). The values of all studied parameters of the assimilatory organs are based on the dry weight.

### 2.4. Data Analyses

Statistical evaluation of the data was performed in the Statistica 9 program (StatSoft, 2008). Results were expressed as mean ± standard deviation (mean ± SD). PCA analysis was realized in the PAST program (version 4.03). Based on the normality of the data distribution, parametric (ANOVA) and non-parametric (Kruskal-Wallis) statistical tests were applied. The Kruskal-Wallis test was used only for Ni content in the assimilatory organs of woody plants. Differences between means were considered significant when they occurred at *p* < 0.05. The Fisher–LSD test was used for pairwise comparisons. Principal component analysis (PCA) was performed using ten selected variables to determine the effective ecological factors concerning specific plant species. Associations between plant and selected soil characteristics were subsequently tested using Pearson’s correlation analysis. These trends were considered statistically significant at *p* < 0.05. Soil contamination factors (CF) were calculated by dividing the heavy metal concentration in the soil by the background value reported by Kabata-Pendias [34].

## 3. Results

### 3.1. Soil Properties

The values of soil analyses are shown in Table 2. The Fluvisol, located in Levice park, is neutral throughout its profile. Carbonate content, expressed in CCE (calcium carbonate equivalent), ranges from 1.2% to 2.4%. It is soil with medium humus content and C/N values indicating high-quality humus.

The Fluvisol located in Želiezovce park is neutral only in the upper 0–10 cm layer. In the lower part, it is slightly alkaline. The CCE ranges from 1.5 to 2.5%. This moderate humus soil has a favorable C/N ratio, lower than 10.6.

The Phaeozem located in Ondrejovce park is neutral, in a depth of 20–30 cm, slightly alkaline soil with a carbonate content from 1.9% to 2.9%. It is a medium humus soil in the upper layer of 0–10 cm and a slightly humus soil in the lower part of the profile, with a favorable C/N ratio.

### 3.2. Cadmium Content in Soil Samples

**Figure 2:**
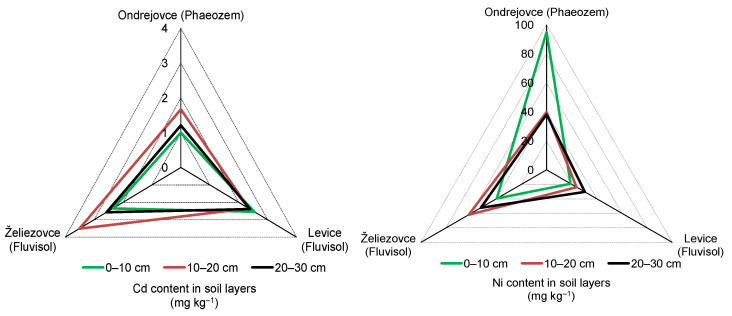
Average content of Cd and Ni in the soil layers of the studied park objects.

The most Cd was accumulated in the upper 0–10 cm layer of Fluvisols in city parks in Levice and Želiezovce (2.4 ± 0.4–2.5 ± 0.4 mg kg^−1^), Figure 2. In lower layers, Cd values varied from 2.4 ± 0.4 to 3.5 ±0.6 mg kg^−1^. The amounts of Cd detected in the Phaeozem of Ondrejovce park fluctuated from 1 ± 0.2 to 1.7 ± 0.3 mg kg^−1^. The average Cd contents in the soil profiles of studied parks declined as follows (mg kg^−1^): Želiezovce (2.8 ± 0.6) ˃ Levice (2.4 ± 0.1) ˃ Ondrejovce (1.3 ± 0.3). Concentrations in city park Levice were slightly variable (with a coefficient variation of 4.1%). Higher dispersion of Cd in soil profiles was observed in Ondrejovce and Želiezovce parks (26% and 22%, respectively).

The values of contamination factors (Table 3) indicated considerable soil Cd contamination in Ondrejovce and Levice parks. In the case of Želiezovce park, they point to very high Cd contamination.

### 3.3. Nickel Content in Soil Samples

Nickel content ranged from 19.1 ± 1.7 to 94.9 ± 8.5 mg kg^−1^ in 0–10 cm layer and from 23.9 ± 2.2 to 62 ± 5.6 mg kg^−1^ in lower layers of soils (Figure 2). The highest Ni content—2.5 times higher compared to lower soil layers—was found in the surface layer of Phaeozem in Ondrejovce park. On the other hand, 1.6 times more Ni was found in lower layers of Fluvisols in Želiezovce and Levice parks than in the surface layer. According to decreasing average amount of Ni in the soil layers (0–30 cm), the parks can be arranged in the following order (mg kg^−1^): Ondrejovce (57.63 ± 32.3) ˃ Želiezovce (51.3 ± 11.2) ˃ Levice (24.47 ± 5.7). The amounts of Ni detected in the soil layers of parks Ondrejovce and Želiezovce are relatively high and indicate Ni contamination. CF values of the moderate Ni contamination of the soils in Ondrejovce and Želiezovce parks and low contamination of the soil in Levice park (Table 3) point to the negative impact of anthropogenic activities in the area of interest.

### 3.4. Cadmium Content in the Assimilatory Organs of Woody Plants

**Figure 3:**
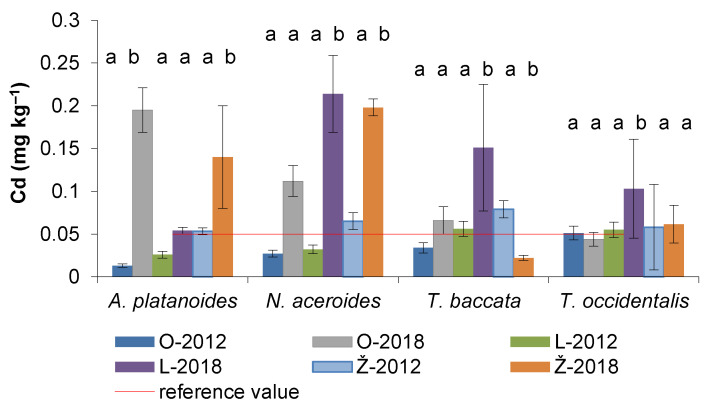
Cd contents (mean ± SD) in the assimilatory organs of woody species in park objects (O–Ondrejovce, L–Levice, Ž–Želiezovce). Different letters (a,b) indicate significantly different Cd values found in 2012 and 2018 in the same park (ANOVA, Fisher–LSD test). The reference value of Markert [36] for plants is 0.05 mg kg^−1^.

In 2012 and 2018, the content of Cd absorbed by the assimilatory organs of the examined woody plants ranged between 0.01–0.08 mg kg^−1^, respectively 0.02–0.22 mg kg^−1^ (Figure 3). The leaves of *A. platanoides* in Ondrejovce and Želiezovce parks showed a several-fold increase in Cd in 2018 compared to 2012. The leaves of *N. aceroides* accumulated significantly more Cd in Levice and Želiezovce parks, while the needles of *T. baccata* and *T. occidentalis* in Levice park. It is interesting that in Želiezovce Park, a significant decrease of Cd in the needles of *T. baccata* was recorded in 2018 compared to 2012 (Figure 3).

**Figure 4:**
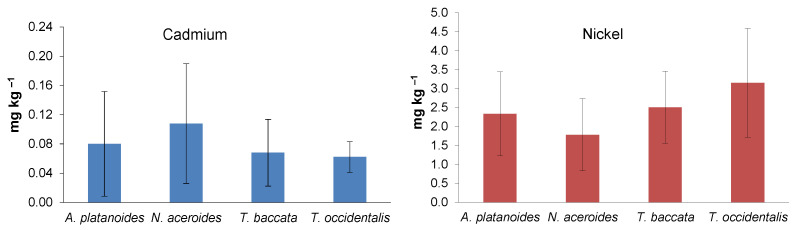
Average contents of Cd and Ni (mean ± SD) in assimilatory organs of woody species for the years 2012 and 2018. The differences between woody species are insignificant (ANOVA, Fisher–LSD test).

Average concentrations of Cd (mg kg^−1^) in the assimilatory organs of conifers in 2012 and 2018 were quite similar: *T. baccata* (0.07 ± 0.04) ˃ *T. occidentalis* (0.06 ± 0.02). In the case of leafy species, Cd content was insignificantly higher in *N. aceroides* (0.11 ± 0.08) compared to *A. platanoides* (0.08 ± 0.07) (Figure 4).

### 3.5. Nickel Content in the Assimilatory Organs of Woody Plants

**Figure 5:**
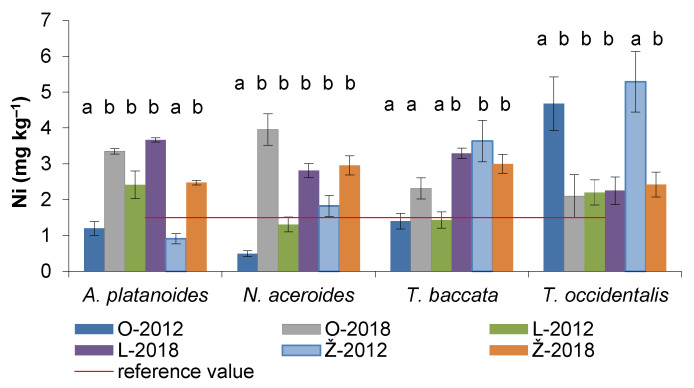
Ni contents (mean ± SD) in the assimilatory organs of woody species in park objects (O–Ondrejovce, L–Levice, Ž–Želiezovce). Different letters (a,b) indicate significantly different Ni values found in 2012 and 2018 in the same park (ANOVA, Fisher–LSD test). The reference value of Markert [36] for plants is 1.5 mg kg^−1^.

The content of Ni in assimilatory organs ranged between 0.5–5.3 mg kg^−1^ in 2012 and between 2.1–3.9 mg kg^−1^ in 2018 (Figure 5). The results showed that Ni in leaves was significantly affected by environmental factors. A significant increase of Ni in 2018 compared to 2012 showed leaves of *A. platanoides* in the parks of Ondrejovce and Želiezovce. A significant increase also showed leaves of *N. aceroides* in Ondrejovce. The needles of *T. baccata* showed a greater ability to accumulate Ni in Levice park, while in Želiezovce park, we even recorded a slight decrease in Ni in 2018 (17%) compared to 2012. A similar trend was also observed for assimilatory organs of *T. occidentalis* in the parks of Ondrejovce and Želiezovce. The decrease of Ni in both cases was 2.2-fold.

The average concentration of Ni (mg kg^−1^) in the assimilatory organs of *T. baccata* (2.51 ± 0.95) was insignificantly lower in the years of observation compared to *T. occidentalis* (3.16 ± 1.43). In the case of leafy species, Ni content was insignificantly higher in *A. platanoides* (2.33 ± 1.10) compared to *N. aceroides* (1.78 ± 0.95) (Figure 4).

### 3.6. Energy Content in the Assimilatory Organs of Woody Plants

**Figure 6:**
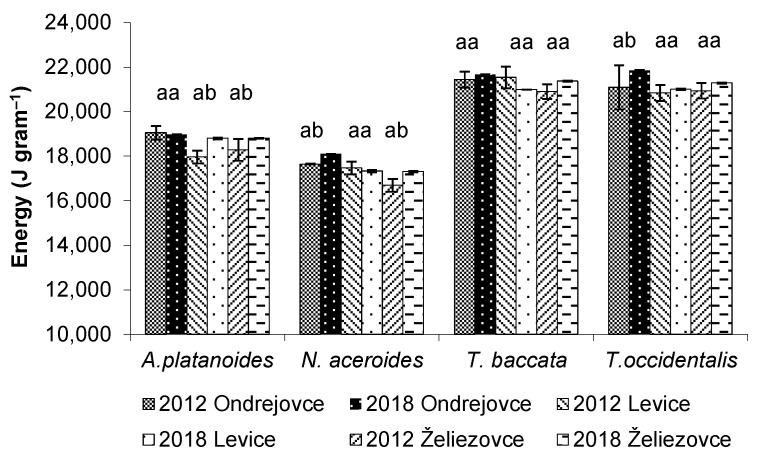
Differences in energy contents ( ± SD) of assimilatory woody species in 2012 and 2018 (O—Ondrejovce, L—Levice, Ž—Želiezovce). Different letters (a,b) indicate significantly different mean values (ANOVA, Fisher–LSD test).

The energy content of leafy woody plants was lower compared to conifers in both years of observation (Figure 6). In 2018, the energy content in the leaves of *A. platanoides* located in Levice and Želiezovce parks and in the leaves of *N. aceroides* located in Ondrejovce and Želiezovce parks increased significantly. A significant increase in energy in woody plants may have been a response to environmental stress, as plants exposed to adverse factors tend to increase energy content.

### 3.7. Principal Component Analysis (PCA)

**Figure 7:**
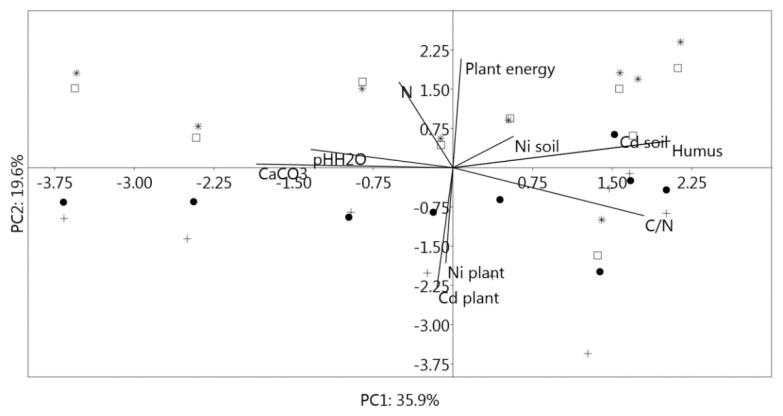
Ordinary diagram with ten variables in the space of the first two principal components; *A. platanoides*—dot; *N. aceroides*—plus; *T. baccata*—square; *T. occidentalis*—star.

Principal component analysis (PCA) was used to visually illustrate the main factors in the multivariate space (Figure 7). Most of the variance was accounted for by the first three components (PC1-PC3; PC1 explains 35.9%, PC2 19.6%, PC3 16.4% of the variance). In total, the first three PCs explained about 72% of the total variability. It means that 72% of the total variance in 10 considered variables can be condensed into three new variables (PCs). The following variables were analyzed: the content of Ni, Cd, and energy in assimilatory organs of woody plants and the content of Ni, Cd, equivalent CaCO_3_, N, humus, pH_H2O,_ and C/N values in soils. Variables are displayed based on the correlation with the main components. So, the first PC is positively correlated with the content of Cd in soil (PC1 0.49033) and the content of humus in soil (PC1 0.49033). These variables are located on the right in the loading plot near the first principal component. Thus, the content of humus in soil is closely related to the transfer of Cd in soil. The content of Cd in the assimilatory organs of the woody plants (−0.55025) located on the left in the loading graph is negatively correlated with the PC2, while energy content in the assimilatory organs of woody plants (0.49836) is positively correlated. Finally, soil Ni content (0.62643) and soil pH_H2O_ values (0.51031) are significant for the PC3.

### 3.8. Relationships between Plant and Soil Characteristics

Table 4 summarizes the results of the correlation analysis of plant and soil characteristics. There is a statistically significant correlation between Ni content in soil and in the leaves of *N. aceroides* (0.662). On the other hand, the correlation coefficient between Ni content in the soil and the needles of *T. baccata* is negative (−0.445). The correlations between Cd contents in assimilatory organs and soils were negative. This means that Cd content in the soil alone cannot reliably indicate the availability of Cd for plants.

The closest negative correlation (−0.655) was found between Ni content in leaves of *A. platanoides* and soil pH value. A strong negative correlation also exists between soil pH value and Cd content in *T. baccata* needles (−0.645). Of the other soil properties, the CaCO_3_ equivalent had the closest negative relationship (−0.553) with Ni content in *T. baccata* needles. On the other hand, CaCO_3_ equivalent value increased Ni content in *N. aceroides* leaves (0.424) and Cd content in *A. platanoides* leaves (0.526). A positive correlation (0.596) was found between soil pH value and the content of Cd in *A. platanoides* leaves.

The total N content in the soil significantly affected Ni content (−0.533) in *T. occidentalis* needles. Negative correlations (−0.846 and −0.708) were also found between the content of N in soil and Cd content in non-native woody plants (*N. aceroides* and *T. occidentalis*). The C/N ratio was most closely related to Cd concentration in *N. aceroides* leaves (0.506) and Ni content in *T. baccata* needles (0.546). Positive correlations (0.763 and 0.589) were also found between the content of Cd and energy in the assimilatory organs of native woody plants (*A. platanoides* and *T. baccata*). The closest relationship was found between Ni content in the soil and the energy content of *N. aceroides* leaves (0.505).

## 4. Discussion

Metal uptake by plants can be affected by several factors, including metal content in soils, soil pH value, cation exchange capacity, organic matter content, species of plant, and plant age [37]. For example, soil pH values negatively affected the distribution of trace metals in the soils of the Sundarbans mangrove areas (Bangladesh) and promoted an inverse interaction between trace metals and soil pH [38]. In general, plants grown in neutral and alkaline soils can uptake less Cd than plants grown in acidic soils. Increasing soil pH from 5.5 to 7.0 significantly decreased Cd concentrations in clover, lettuce, carrot, and ryegrass, and to a lesser extent in wheat [39]. According to Wolińska et al. [40], soil organic matter participates in the retention, reduction of mobility, and bioavailability of HM. We found the highest soil Cd content in combination with the medium content of organic matter (3.8 to 4.2%) in Levice and Želiezovce parks. However, it must be said that these parks are located near busy roads with high emission production. Significantly less soil Cd was recorded in Ondrejovce park, located at a greater distance from important traffic routes. Al-Taani et al. [41] reported that high Cd content in soil might be related to multiple sources, including agrochemicals, atmospheric dust containing HM, and traffic-related metal emissions.

According to Uminska [42], Cd content in soils in excess of 1 mg kg^−1^ is considered to be evidence of anthropogenic pollution. Overall, the concentrations of Cd in the soils of studied parks were relatively high, 3–7 times higher than the global soil average (0.41 mg kg^−1^) stated by Kabata-Pendias [34]. Cadmium content in soils was also markedly higher than the median value of 0.3 mg Cd kg^−1^ given by Čurlík and Šefčík [43] for Slovak soils. Fazekašová et al. [44] found an above-limit content of Cd (9.6 mg kg^−1^) in soils located in the vicinity of Sereď nickel smelter (SW Slovakia). The average concentrations of Cd found in the soils of the investigated urban parks are even higher than the value of 1.58 mg kg^−1^ determined in the soils of several Chinese cities by Wei and Yang [3]. Based on the pollution index, the aforementioned authors state that in China’s industrial cities, soil and road dust are highly contaminated with HM from anthropogenic activities, especially from transportation and industrial sources.

The average amounts of Ni detected in the soil profiles of Ondrejovce and Želiezovce parks were significantly different compared to the average Cd in the soil of Levice park. Nickel content fluctuated the least in the soil of Želiezovce park (coefficient of variation of 22%) and most in the soil of Ondrejovce park (coefficient of variation of 56%). This means that the spatial distribution of Ni in the soil of Ondrejovce park was not homogeneous. The average concentrations of Ni found in the soils of investigated urban parks are lower than the value of 99.48 mg kg^−1^ determined in the soils of several Chinese cities by Wei and Yang [3]. On the other hand, the soils in the studied parks are more contaminated with Ni than soils in Chinaʼs parks, for which Chen et al. [45] report a value of 22.2 ± 8.7 mg kg^−1^. The characteristic interval for uncontaminated Slovak soils represents 18–34 mg kg^−1^ [46]. In world soils, the content of Ni ranges from 13–37 mg kg^−1^. Higher concentrations of Ni in soil layers may reflect the influence of both soil-forming processes as well as anthropogenic activities [34]. Contamination factor values determined in the soils of investigated parks indicate low to moderate Ni pollution, similar to that reported by Al-Taani [41] from the Liwa area (1.64 to 2.61) in the United Arab Emirates. Kirkham [47] reports that most plants growing under polluted conditions show Cd concentration ≥0.1 mg kg^−1^. Ladipo et al. [48] report Cd concentration in leafy vegetables from 0.028 to 0.091 mg kg^−1^ dry weight. In park objects, the assimilatory organs of woody plants, in most cases, had Cd concentrations > 0.05 mg kg^−1^, which is the reference value in plants reported by Markert [36].

Cadmium content in studied parks reached 0.6–0.7 mg kg^−1^ in coniferous and 0.7–0.11 mg kg^−1^ in leafy woody species. The lowest content was found in the needles of *T. occidentalis* and the highest in the leaves of *N. aceroides*. Differences in Cd values point to different accumulative abilities of woody plants. Liu et al. [49] also report that plants can readily respond to the amount of a readily mobile metal in the soil. Molnárová et al. [50], for example, report that leaves of the *Salix* species in the Malé Karpaty Mountains (SW Slovakia) had high Cd levels (0.06–0.36 mg kg^−1^), while Cd content in the leaves of *Alnus glutinosa* is lower (0.01–0.18 mg kg^−1^). Cadmium was the most bioavailable metal in soils in areas contaminated by slag from an abandoned steel plant in Havana, reflected in the high accumulation of Cd (1.0 mg kg^–1^) in leafy vegetables [51]. According to Ismael et al. [52], higher values in plants can be explained by the greater efficiency of plants in absorbing metal from soil and air at contaminated sites. In studied parks with high anthropogenic load, several times higher (7–128) contents of Cd were found in soils compared to assimilatory organs of woody plants. In city parks of Central Bohemia with a low anthropogenic load, there was only 1.8 times more Cd in soils than in the leaves of the *Taraxacum* sp. In the area of the dominant industrial enterprise, the authors found 3.1 times more Cd in soils than in plants [53].

Nickel is found in the vegetative organs of most plants in the range of 1–10 mg kg^−1^ [54]. According to Agyarko et al. [55], it becomes toxic to plants when absorbed beyond the normal ranges of 0.02–5 mg kg^−1^. However, some plant species may not absorb Ni in their tissues, although the measured concentration of Ni in soil samples can be high [56]. As a result of environmental pollution, Ni content in the studied woody plants has increased considerably. In coniferous woody plants, the average value of Ni was 27% higher than in deciduous ones. In study parks, several times higher Ni (7–21) contents were found in the soils compared to plants. Compared to our results, Fröhlichová et al. [53] recorded up to 25 times higher Ni content in soils than in *Taraxacum* leaves growing in city parks of Central Bohemia with low anthropogenic loads. In the area of the dominant industrial enterprise, there was even up to 58 times more Ni in the soils than in the plants. However, it should be noted that surface-contaminated soil organic matter does not control Cd uptake, as plant roots can grow into uncontaminated subsoil [57].

The energy content in needles (20,800–21,846 J g^−1^) was higher compared to the energy content in the leaves (16,688–19,051 J g^−1^) of park woody plants. The assimilatory organs of coniferous species had similar energy contents in the study period and are more tolerant to pollution. The energy values found in the leaves of park woody plants are similar to those reported by other authors. Pňakovič and Dzurenda [58] report the values of heat of combustion of fallen leaves of several deciduous trees in the range of 16,046–20,247 J g^−1^. Nurmi [59] determined the calorific values of spruce needles in the range of 19,224–19,298 J g^−1^. The calorific value of pine needles determined by Font et al. [60] reaches 20,140 J g^−1^. Kelsey et al. [61] report 20,240 J g^−1^ for *Larix occidentalis* needles and up to 22,400 J g^−1^ for *Thuja plicata* needles.

The results of our research point to synergistic effects, as a result of which the content of Cd or Ni in the leaves of woody plants can increase or decrease dependent on soil properties. The closest negative correlation in relation to soil pH was shown by Ni content in the leaves of *A. platanoides.* There is also a strong negative correlation between soil pH value and Cd content in the needles of *T. baccata.* Soil pH value and clay content determine the solubility of metals in the soil and their availability for plant uptake [62]. The authors observed a strong correlation between soil pH value and Cd and Ni concentrations in all vegetables. Hinesly et al. [63] report that soil pH value can greatly affect Cd uptake and transportation in corn. The plant uptake of the elements generally decreases as soil pH increases. Overall, assimilatory organs of park woody plants had higher Cd concentrations despite the fact that they grew on neutral to slightly alkaline soils. A significant positive correlation was found between soil pH value and Cd content in *A. platanoides* leaves. Chiy and Phillips [64] reported that positive correlation coefficients between soil pH values and Cd contents in the ryegrass could be derived from ionic competition when the content of Na can increase Cd in the plant.

The bioavailability of Ni in plants can also vary with soil properties [65]. Soil Ni content, cation exchange capacity, and nitrogen fertilizer amount are the three most critical variables for modeling Ni bioaccumulation in the soil–rice system [66]. Alegría et al. [67] found the highest correlation between Ni content in soil and vegetables. The mobile forms of nickel in oil-contaminated soils in the Precarpathians of Ukraine had the maximum synergistic effect on the absorption of Cd and Ni by *Silphium Perfoliatum* roots [68]. In studied parks, in turn, a significant correlation was found between Ni content in the soil and its content in *N. aceroides* leaves. On the other hand, the correlation between Ni content in the soil and the needles of *T. baccata* was negative, and in the case of *A. platanoides* and *T. occidentalis,* the correlation coefficients were insignificantly negative. This means that the soil Ni concentration alone does not indicate the availability of Ni for these woody species. The relationships of HM in rice with those in soil, along with soil pH value, were well described by linear regression models by Zhao et al. [69]. The authors found an insignificant correlation between the concentrations of Ni in soil and those in rice. Madejón et al. [22] also found that Ni is the element with the lowest correlation coefficients between poplar leaves and soil.

## 5. Conclusions

The research results provide information on the state and development of contamination of two historical urban and one rural park affected by polluted air. It was found that, currently, the concentrations of Cd and Ni in soils and assimilatory organs of woody plants already exceed the background values. Based on the values of the contamination factors, we can conclude that the soils in Ondrejovce and Levice parks are considerably contaminated with Cd. The soil of Želiezovce park is highly contaminated with Cd and moderately contaminated with Ni.

The location of woody species in the multivariate space of PCA showed that the content of soil Cd and humus, as well as the energy content of needles, significantly correlated with coniferous woody species. On the other hand, leaf Cd content is a significant variable for deciduous woody species. The first three principal components significantly correlated with the content of Cd (PC1) and Ni (PC3) in soils and Cd (PC3) content in plants, suggesting that these elements could especially originate from industrial and vehicular sources. The significant negative correlation observed for Cd content in *A. platanoides* and *N. aceroides* leaves with PC2 suggests that soil Cd content was probably not the most important variable for Cd bioaccumulation in leafy species but rather comes from polluted urban air.

The research results point to the importance of the assimilatory organs of woody plants as an important factor in reducing the amount of pollutants in the soil and air of urban and rural settlements. They also contribute to knowledge about the ability of native and non-native woody species to accumulate dangerous metals, tolerate polluted environments, and adapt to changed climatic conditions. In the future, it will be necessary to focus also on some other important pollutants associated with the constantly changing urban and rural environment.

## Figures and Tables

**Table 1:** Basic information about the studied parks.

Park Object	Categorization	Altitude [m]	Geographical Coordinates
Levice city	historical, origin 1879	160	48°12′ N, 18°36′ E
Želiezovce city	historical, origin 1875	145	48°02′ N, 18°39′ E
Ondrejovce village	historical, origin 1900	163	48°08′ N, 18°31′ E

**Table 2:** Soil properties.

Park Object	Soil Unit	Soil Layer	Soil Reaction		CaCO_3_ Equiv.	Humus	Ct	Nt	C/N
		[cm]	[pH_H2O_]	[pH_KCl_]	[%]				
Levice	Pantofluvic Endostagnic Katocalcaric Hypereutric Fluvisol	0–10	6.75	7.20	1.17 ± 0.13	3.81 ± 0.11	2.21 ± 0.10	0.28 ± 0.3	7.9 ± 0.6
		10–20	6.82	7.23	1.51 ± 0.17	2.93 ± 0.09	1.70 ± 0.10	0.13 ± 0.1	12.9 ± 1.0
		20–30	7.09	7.34	2.40 ± 0.26	1.98 ± 0.06	1.15 ± 0.05	0.24 ± 0.2	4.7 ± 0.4
Želiezovce		0–10	7.10	7.15	1.50 ± 0.16	4.12 ± 0.12	2.39 ± 0.11	0.26 ± 0.3	9.3 ± 0.7
		10–20	7.22	7.26	2.10 ± 0.23	3.55 ± 0.11	2.06 ± 0.10	0.21 ± 0.2	10.0 ± 0.8
		20–30	7.25	7.42	2.46 ± 0.27	3.21 ± 0.10	1.86 ± 0.09	0.17 ± 0.2	10.6 ± 0.9
Ondrejovce	Pantohypocalcic Phaeozem (Pantosiltic)	0–10	6.94	7.16	1.90 ± 0.21	4.21 ± 0.13	2.44 ± 0.12	0.24 ± 0.2	10.1 ± 0.8
		10–20	7.10	7.33	1.98 ± 0.22	2.79 ± 0.08	1.62 ± 0.09	0.26 ± 0.1	6.2 ± 0.5
		20–30	7.24	7.38	2.88 ± 0.32	1.72 ± 0.05	1.00 ± 0.04	0.26 ± 0.1	3.7 ± 0.3

Note: Ct—total carbon, Nt—total nitrogen.

**Table 3:** Average Cd and Ni contents and contamination factor (CF) values for the top 30 cm layer of soils. Significantly different values (*p* ˂ 0.05) are indicated by different letters (^a,b^).

Park/Soil Unit	Cadmium			Nickel		
	Average (0–30 cm)	CF ± SD	The Level of Contamination,	Average (0–30 cm)	CF ± SD	The Level of Contamination,
	[mg kg^−1^ ±SD]		Hakanson [35]	[mg kg^−1^ ±SD]		Hakanson [35]
Ondrejovce/ Phaeozem	1.29 ± 0.34 ^a^	3.2 ± 0.8	considerable	57.63 ± 32.3 ^b^	1.9 ± 1.1	moderate
Levice/ Fluvisol	2.43 ± 0.10 ^b^	5.9 ± 0.3		24.47 ± 5.7 ^a^	0.8 ± 0.2	low
Želiezovce/ Fluvisol	2.81 ± 0.62 ^b^	6.9 ± 1.5	very high	51.30 ± 11.2 ^b^	1.8 ± 0.4	moderate

Note: world average [34]—Cd 0.41 mg kg^−1^, Ni 29 mg kg^−1^.

**Table 4:** Correlation analysis of relationships between plant and soil characteristics.

	Characteristic	Ni Content in Plant			Cd Content in Plant		
		F-Ratio	R-Squared (R^2^)	Correlation Coefficient (R)	F-ratio	R-Squared (R^2^)	Correlation Coefficient (R)
*A. platanoides*							
Soil layer 0–30 cm	Ni/Cd	0.877	0.111	−0.334	0.686	0.005	−0.076
	pH_H2O_	5.269	0.429	−0.655 *	3.849	0.355	0.596 *
	CaCO_3_ equivalent	0.143	0.020	−0.142	2.679	0.277	0.526 *
	Humus	1.248	0.151	−0.389	0.041	0.006	−0.077
	N	0.082	0.012	0.108	0.231	0.032	0.179
	C/N	0.577	0.076	−0.276	0.368	0.049	−0.223
	Leaf energy	0.002	0.000	−0.001	9.775	0.583	0.763 **
*N. aceroides*							
Soil layer 0–30 cm	Ni/Cd	5.464	0.438	0.662 *	4.902	0.031	−0.178
	pH_H2O_	0.279	0.038	0.196	0.005	0.0007	0.002
	CaCO_3_ equivalent	1.537	0.180	0.424 *	0.005	0.001	−0.009
	Humus	0.070	0.010	−0.099	0.230	0.032	−0.178
	N	0.358	0.049	0.221	17.59	0.715	−0.846 **
	C/N	0.426	0.057	−0.240	2.412	0.256	0.506 *
	Leaf energy	2.392	0.255	0.505 *	0.099	0.014	−0.118
*T. baccata*							
Soil layer 0–30 cm	Ni/Cd	1.731	0.198	−0.445 *	0.073	0.120	−0.347
	pH_H2O_	0.735	0.095	−0.308	4.989	0.416	−0.645 *
	CaCO_3_ equivalent	3.137	0.309	−0.556 *	0.399	0.054	−0.232
	Humus	0.960	0.121	0.347	0.957	0.120	−0.347
	N	1.231	0.149	−0.387	0.942	0.119	−0.344
	C/N	2.979	0.299	0.546 *	0.050	0.007	0.084
	Needle energy	0.136	0.020	0.138	3.697	0.356	0.589 *
*T. occidentalis*							
Soil layer 0–30 cm	Ni/Cd	0.866	0.110	−0.331	1.635	0.084	−0.289
	pH_H2O_	1.079	0.134	0.365	0.962	0.121	−0.348
	CaCO_3_ equivalent	0.111	0.016	0.125	0.237	0.033	−0.181
	Humus	0.620	0.081	−0.285	0.639	0.084	−0.289
	N	2.777	0.284	−0.533 *	7.022	0.501	−0.708 **
	C/N	0.279	0.038	0.196	1.073	0.133	0.364
	Needle energy	0.376	0.051	0.226	0.008	0.0001	−0.011

Note: * significant correlation at *p* < 0.05; ** significant correlation at *p* < 0.01.

## Data Availability

Not applicable.
